# Assessment of the residual bleeding burden in hemophilia B on factor IX prophylaxis

**DOI:** 10.1016/j.rpth.2026.107007

**Published:** 2026-10-09

**Authors:** Wolfgang Miesbach

**Affiliations:** University Hospital Frankfurt, Frankfurt, Germany

Hemophilia B affects roughly 1 in 5 people with hemophilia and has long been overshadowed by hemophilia A, with fewer and smaller trials and evidence borrowed from a disease with a different factor and half-life [1]. Whether the bleeding phenotype also differs has been debated since registry analyses showed less joint disease in hemophilia B [2]. Severe hemophilia B carries a higher proportion of non-null mutations with residual factor synthesis [3], and factor IX (FIX) distributes beyond the circulation into an extravascular compartment that contributes to hemostasis at plasma levels which would appear insufficient [4]. Patients with severe hemophilia A have about a 3-fold higher risk of joint arthroplasty [5], yet in young children followed prospectively the 2 diseases bleed alike [6]. What has been missing is a like-for-like estimate of how much bleeding remains once prophylaxis is in place. Lindsley and colleagues now fill that gap in an unusually thorough systematic review and meta-analysis [7].

The review identified 105 studies published between 2005 and 2025, of which 77 entered the meta-analysis: 24 clinical trials and 53 observational studies, the latter prospective or retrospective cohort studies. Every participant was on prophylactic FIX replacement, standard or extended half-life; gene therapy and rebalancing-agent studies contributed only their lead-in periods on FIX prophylaxis. Patients of any severity without inhibitors were eligible, including children and adults, and the main analyses pool 965 patients from trials and 2737 from observational studies.

Each reported annualized bleeding rate (ABR) was classified according to what it counted, where specified in the publication: total for treated and untreated bleeds together, treated for bleeds that received FIX, and unspecified otherwise. The main result was a treated ABR of 2.23 (95% CI 1.52–3.27) across 13 trials and 547 patients; with unspecified estimates added, it was 2.25 (1.67–3.03) in 22 trials and 2.08 (1.62–2.66) in 48 observational studies. Freedom from treated or unspecified bleeding was reported for 29.1% of participants in 7 trials and 47.2% in 16 observational studies, over median follow-up of 27 and 12.3 months; 11 of those 16 studies did not report it.

Two earlier reviews took the same approach. In hemophilia B, Franchini and colleagues in 2025 reviewed 42 studies, 2 of them randomized, and pooled 37 cohorts with 1545 observations: mean ABR 1.29 (0.91–1.66) with extended half-life products (732 observations) against 3.12 (2.63–3.62) with standard half-life (786), and, across 466 observations, zero bleeding in 53% against 24% [8]. The present subgroup estimates, 1.37 and 3.58, fall close to those values. In hemophilia A, a review of 58 publications on prophylaxis with factor VIII (FVIII)-containing products, plasma-derived and recombinant, standard and extended half-life, reported a pooled ABR of 3.4 (3.0–3.7) with 38.5% bleed-free across 48 interventional studies, and 4.8 (4.0–5.5) with 21.8% bleed-free across 10 observational studies [9]. Neither review states how many individual patients underlie its estimates; with 965 and 2737, the present one does.

The comparison between the 2 diseases requires 2 qualifications. The hemophilia A figures cover FVIII replacement including extended half-life concentrates, but predate both the general use of emicizumab and the arrival of efanesoctocog alfa, so they describe the factor era rather than current practice; and the bleed-free proportions diverge in opposite directions, which more plausibly reflects era, definition, and follow-up than the diseases. The mean rates, though, are consistent with the milder phenotype long attributed to hemophilia B: about 2 treated bleeds a year under FIX prophylaxis against 3 to 5 under FVIII. With standard half-life FIX, however, rates of 3.58 in 2 trials and 5.10 in observational studies were no lower than under FVIII. Importantly, a milder phenotype has not produced freedom from bleeding for most patients.

8Asked a decade ago, patients wanted fewer infusions and better efficacy from new concentrates [10]; the aim is now freedom from bleeding, and only a pooled benchmark can show how far FIX prophylaxis meets it. The table sets it beside the 2 programs reporting hemophilia B separately, concizumab [11] and etranacogene dezaparvovec [12]. Marstacimab, licensed for hemophilia B without inhibitors since 2024, and fitusiran, licensed in the United States since 2025, do report estimates by disease type, but only as small subgroups of trials that pooled both diseases, with wide confidence intervals; in BASIS they appear only in a supplementary figure [13], [14]. Neither can carry a comparison.

Such comparisons are valid only when 2 conditions are met; both are routinely overlooked. Count the same bleeds: HOPE-B counted all bleeding episodes at any site, treated or not, and untreated bleeds were 1 in 8 during its lead-in on FIX prophylaxis but 1 in 3 after gene therapy [12]. Observe for the same time: treated ABR in the trials here fell from 2.97 to 1.73 once observation passed 12 months, whereas the proportion free of bleeding falls as observation lengthens.

Freedom from bleeding, rather than the mean rate, separates the options most clearly. Under FIX prophylaxis, fewer than half of patients were free of bleeding over a median of 12 to 27 months, and under a third in the trials; after gene therapy, at least two-thirds were free of any bleeding in every 6 months, against 26% of the same patients during the lead-in, and bleeding rates stayed low for 5 years (Table) [12], [15]. Concizumab reports no bleed-free proportion for hemophilia B. The comparison is indirect, and the gene therapy cohort was small, adult, and selected for a severe bleeding phenotype, but it is the only evidence in this literature that zero bleeds is within reach for the majority, and it comes from gene transfer rather than from intensifying factor replacement.

**Table:** Bleeding on factor IX prophylaxis and on the 2 options with hemophilia B-specific results.

	Concizumab (explorer8) [11]	Etranacogene dezaparvovec (HOPE-B) [12], [15]	FIX prophylaxis (present meta-analysis) [7]
Hemophilia B participants	66 in the trial; 36 in the randomized comparison (12 no prophylaxis, 24 concizumab)	54	965 (trials); 2737 (observational); extended half-life 255 and 1878, standard half-life 86 and 381
ABR on treatment	3.1, treated	0.99, treated (months 7–24); 1.52, total (months 7–60)	2.23, treated (trials); 2.08, treated or unspecified (observational)
ABR in the comparator	14.8, treated	3.65, treated; 4.16, total (lead-in)	Not applicable
ABR by-product generation	Not applicable	Not applicable	Extended half-life 1.37, standard half-life 3.58 (trials); 1.70 and 5.10 (observational)
Participants free of bleeding	Not reported for hemophilia B separately	At least 67% in every 6 months after gene therapy, any bleed, against 26% during lead-in	29.1% (trials); 47.2% (observational), treated or unspecified bleeds
Comparator	No prophylaxis (on-demand treatment), randomized	Lead-in on FIX prophylaxis	None; pooled estimate

Subgroup ABRs by product generation use the same numerators as the overall estimates. Bleed-free proportions in the meta-analysis refer to median follow-up of 27 months (trials) and 12.3 months (observational studies).

ABR, annualized bleeding rate (means); FIX, factor IX.

The comparison is also sensitive to which generation of FIX product stands on the other side. The 3 licensed extended half-life concentrates, albutrepenonacog alfa, nonacog beta pegol, and eftrenonacog alfa, form the extended half-life subgroup of this analysis, where ABR was 1.37 in trials and 1.70 in observational studies. Measured against these figures rather than the overall 2.2, the distance to the newer agents narrows or disappears: gene therapy, at 0.99, stays below them; concizumab, at 3.1, does not. The gain from extending half-life is also specific to hemophilia B: in hemophilia A the pooled ABR was 3.5 with standard and 3.1 with extended half-life FVIII, a difference of no consequence, whereas in hemophilia B it is 2-fold to 3-fold [8], [9]. The simplest explanation is pharmacokinetic: half-life extension reaches 3-fold to more than 5-fold for FIX but only 1.4-fold to 1.6-fold for FVIII, whose clearance is tied to von Willebrand factor [16]; extravascular FIX may contribute [4], another reason to compare the 2 diseases with caution.

Which bleeds are counted matters as much as which comparator is chosen. In this analysis, only 2 of the 24 trials reported total ABR, both lead-in phases of gene therapy studies, and no observational study did. Whether treated and untreated bleeds differ materially in the era of factor replacement therefore remains open. The standards are not new. In 2014, the International Society on Thrombosis and Haemostasis Scientific and Standardization Committee (SSC) defined the bleeding episode, the joint bleed, and the target joint [17]; the World Federation of Hemophilia guidelines call for bleeding outcomes to be reported with their definitions [16]; the coreHEM consortium, convened with the National Bleeding Disorders Foundation and patient organizations, set a core outcome set for gene therapy in hemophilia [18]; and the SSC recommendations published this year go further, making aura an optional criterion for a joint bleed and recommending that the total bleed rate, not the treated rate, serve as the index parameter for efficacy, that the efficacy period be at least 12 months, that zero-bleed rates always carry their observation period, and that women with hemophilia be included with menstrual outcomes reported [19]. Ascertainment adds a second layer: diaries, applications, chart review, or registry entry, with differing definitions of a joint bleed and no common threshold for treating on suspicion. Joint imaging was not captured, so subclinical bleeding lies outside every ABR in this literature. Spontaneous and traumatic bleeds contributed almost equally in the trials, 1.20 and 1.24 per year, and prophylaxis exists to abolish the former.

Three gaps in the source material limit what any pooled figure can settle. Severity was not stratified, so moderate and mild disease dilute the estimate for severe hemophilia B, the group in which the comparison with hemophilia A is drawn. Sex is nowhere reported, leaving symptomatic carriers and women invisible. FIX trough levels, regimen intensity, and adherence could not be captured, although the label on the vial acts largely through them; prospective noninterventional studies show what pooled literature cannot [20].

To document the real picture, future studies should distinguish between these outcomes. Report total and treated bleeds separately and define both; name the method of ascertainment; state the observation period and do not calculate ABR over less than 12 months; present mean and median; give zero-bleed proportions with their follow-up; report severity, trough level, and adherence wherever bleeding is reported. Two bleeds a year is the best estimate the field has for hemophilia B under prophylaxis, and this analysis is what makes it usable: it states what was counted, and what was not.
